# Metagenomic Profiling Reveals Extensive Bacterial Diversity in Chicken Manure and Associated Contaminated Wastewater

**DOI:** 10.3390/ijms27093741

**Published:** 2026-04-23

**Authors:** Sadir Zaman, Nawab Ali, Waheed Ullah, Nadia Taimur, Noor ul Akbar, Aiman Waheed, Niaz Muhammad, Muhammad Saeed Khan

**Affiliations:** 1 Department of Microbiology, Kohat University of Science and Technology, Kohat 26000, Pakistan; sadirzaman33@gmail.com (S.Z.);; 2 College of Engineering and Energy, Abdullah Al-Salem University, Khaldiya Campus, Kuwait City 72300, Kuwait; 3 Department of Biotechnology and Genetic Engineering, Kohat University of Science and Technology, Kohat 26000, Pakistan; 4 Department of Botany, Kohat University of Science and Technology, Kohat 26000, Pakistan; 5 Department of Zoology, Kohat University of Science and Technology, Kohat 26000, Pakistan; 6 Centre of Biotechnology and Microbiology, University of Peshawar, Peshawar 25120, Pakistan

**Keywords:** metagenomics, chicken manure, wastewater microbiome, pathogen dissemination

## Abstract

Chicken manure and its potential to contaminate water systems through the dispersal of pathogenic bacteria are major concerns in environmental and public health. In this study, a metagenomic analysis was employed to systematically identify and compare bacterial assemblages in chicken manure (CM) and in a contaminated sample of chicken manure wastewater (CMW). Whole DNA was extracted from CM and CMW, followed by whole-genome shotgun sequencing; data analysis was done using online Galaxy software (ver. 26.0.1.dev1). Metagenomic analysis reveals a complex One Health challenge. Data showed that CM and CMW are different in their microbiota, as indicated by a distinct separation of beta diversity values and limited overlapping of species between sample types. In the current study, we found a greatly significant common functional set of adapted bacterial masses, including major pathogenic bacterial groups as well as opportunistic and environmental bacterial species, indicative of a direct contamination from CM and CMW. Notably, in both CM and CMW, a plethora of opportunistic, enteric, and environmental pathogens like *Escherichia coli*, *Salmonella enterica*, and *Acinetobacter baumannii* were found, coupled with an indication of a direct functional flow between both ecosystems as tangled reservoirs. Chicken manure samples showed differences in taxonomic composition and inferred functional profiles at the time of sampling: CM1 was pathogen-enriched, CM2 exhibited strong nitrogen-supportive metabolism, CM3 was dominated by fiber-degrading decomposers, and CM4 showed high methane-producing potential with environmental risk. Such findings underscore the raising of chickens as a potential source of harmful bacteria for the environment. It is important to note that this study represents a preliminary investigation with certain limitations, including the absence of biological replicates, lack of temporal sampling, and limited capacity to infer dynamic ecological interactions. Yet this metagenomic report is more about describing the taxonomy and functional potential of the bacteria, rather than discussing the actual ecological processes of these microorganisms in the environment. Future studies will be required to explore these aspects.

## 1. Introduction

The poultry industry has developed into a major agro-based sector, contributing significantly to food production, economic growth, and employment. Large-scale production of poultry meat and eggs generates substantial quantities of chicken manure and wastewater. These waste products serve as important reservoirs of diverse microbial communities and potential environmental contaminants, emphasizing the need for comprehensive metagenomic profiling to understand bacterial diversity and associated risks [1]. Organic waste is a by-product that is generated in large amounts; for example, 1000 broilers are estimated to generate approximately 120 kg of feces a year [2]. Chicken litter consists of a complex microbiota derived from gastrointestinal and environmental sources [3,4,5]. Microbial profiling shows that the avian gastrointestinal tract is dominated by the phyla Proteobacteria and Bacteroidetes, and there is often also a large presence of Firmicutes in fecal samples, which can be larger in captive poultry in comparison to wild birds [6,7]. Improper disposal of this waste can lead to the contamination and eutrophication of water bodies [1,8]. Bacterial communities in chicken manure wastewater (CMW) include ammonia oxidizers (*Nitrosomonas europaea*, *Nitrobacter winogradskyi*), floc formers (*Zoogloea ramigera*), and degraders (Pseudomonas aeruginosa). Many bacteria, including fermenters (*Clostridium perfringens*, *Bacillus subtilis*) and intestinal microbes (*Bacteroides fragilis*), are shared between chicken manure and wastewater due to direct inoculation from manure runoff, which introduces bacteria pre-adapted to degrade these compounds (as demonstrated in the study titled “Metagenomic Profiling Reveals Extensive Bacterial Diversity in Chicken Manure and Associated Contaminated Wastewater”) [9].

Poultry litter is a significant source of microbiological and chemical contamination in soil. In addition to introducing diverse microorganisms and potential pollutants, it alters key physicochemical properties such as pH, organic carbon, and available nitrogen. These changes can disrupt the native soil microbial community structure and influence overall soil ecosystem functioning [10]. Environmental dissemination of poultry waste through soil leaching and surface runoff serves as a major pathway for contamination of nearby water bodies. Metagenomic analysis of contaminated wastewater reveals high bacterial diversity originating from manure, indicating an efficient mechanism for the environmental spread of microorganisms. This distribution also facilitates the transfer of genetic elements, including antibiotic resistance genes, thereby raising concerns about microbial pollution and public health risks associated with contaminated water systems [11]. Intensive poultry farming is often associated with substantial disease burdens, which can lead to significant production losses due to infectious pathogens such as Avian influenza and Newcastle disease [12,13,14]. As a strategy to prevent and control these disease outbreaks, the prophylactic and therapeutic use of antimicrobials became widespread in intensive poultry production systems [15]; therefore, the persistent use of antibiotics leads to the global threat of antimicrobial resistance (AMR) [16].

From a One Health perspective, metagenomic profiling has identified poultry manure and contaminated wastewater as significant reservoirs of diverse bacterial communities with implications for animal, environmental, and human health. Shotgun metagenomic analysis has revolutionized culture-independent approaches for investigating complex microbial ecosystems by enabling comprehensive identification and characterization of all genetic material within a sample, including protein-coding genes and antimicrobial resistance determinants. This untargeted approach provides an integrated view of taxonomic composition and functional potential, supporting the One Health framework by linking microbial diversity, environmental dissemination, and potential public health risks across interconnected ecosystems [17,18].

The current study aims to explore bacterial species that are diverse due to the lack of a hygienic environment, as well as the exposure of poultry to diverse bacterial pathogens. Bacteria associated with human interaction have a higher chance of disseminating the bacterial species to both the community and the environment. By examining both the primary source and its environmental transmission pathway, the study investigates how diverse microorganisms, including potential pathogens and resistance genes, disseminate across ecosystems. Within a One Health framework, this approach links poultry waste, environmental contamination, and potential human exposure, emphasizing the interconnected nature of animal, environmental, and public health.

## 2. Results

### 2.1. Bacterial Community Structure in Chicken Manure (CM)

**Figure 1:**
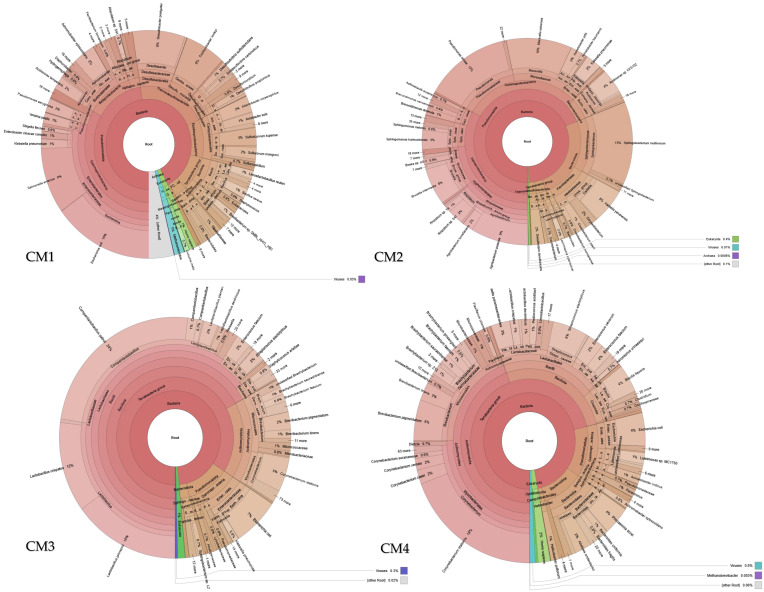
Krona plots showing taxonomic hierarchy from phylum (inner ring) to species (outer ring) for CM1, CM2, CM3, and CM4. Colored sectors represent relative abundance.

In CM1, the outermost ring of the Krona plot shows *Salmonella enterica* and *Escherichia coli* as detectable taxa. In CM4, *Clostridium perfringens* appears as a distinct sector. In CM2 and CM3, no enteric pathogens are visible at the species level; instead, *Lactobacillus* spp. and *Bacteroides* spp. dominate the outer rings. Their presence underscores the importance of monitoring, managing, and mitigating microbial hazards in poultry manure and associated wastewater to safeguard public health and maintain ecosystem integrity (Figure 1).

### 2.2. Bacterial Community Structure Krona Plot Analysis of Chicken-Manure-Contaminated Wastewater Samples

**Figure 2:**
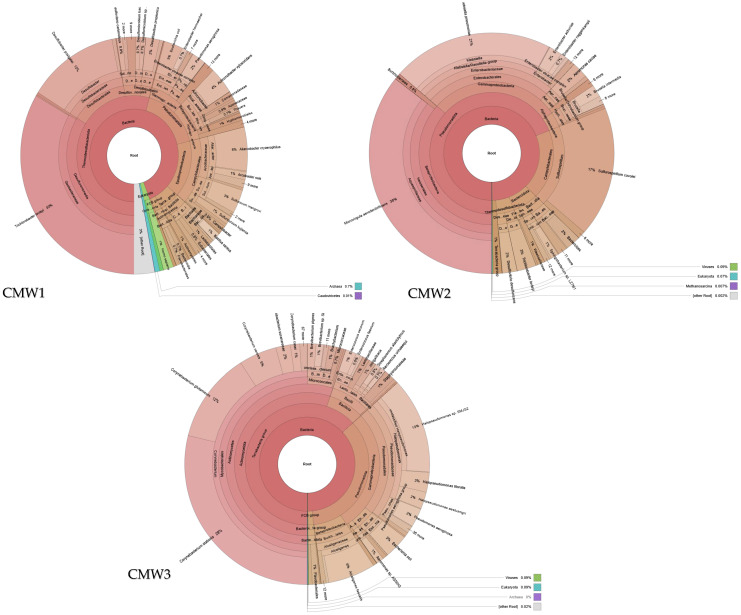
Krona plots depicting the taxonomic hierarchy from phylum (inner ring) to species (outer ring) for specimens CMW1, CMW2, and CMW3. Colored sectors indicate relative taxonomic abundance at each rank.

In CMW1, CMW2, and CMW3, the inner ring of each Krona plot is dominated by the domain Bacteria. The phylum-level ring shows Firmicutes (brown sectors), Bacteroidetes (red-brown), and Proteobacteria (green) in all three samples. At the genus level, CMW1 shows *Nitrosomonas* and *Nitrobacter*; CMW2 shows *Pseudomonas* and *Clostridium*; and CMW3 shows *Bacillus* and *Bacteroides*. Species-level resolution identifies *Nitrosomonas europaea* in CMW1, *Pseudomonas aeruginosa* in CMW2, and *Bacillus subtilis* in CMW3. However, differences in sector sizes and labels at the genus and species levels indicate that specific taxa vary among CMW1, CMW2, and CMW3, suggesting local variation in contamination sources, nutrient loads, or treatment conditions. Importantly, several of these taxa include potential pathogens or opportunistic bacteria, highlighting the risk of microbial dissemination from poultry waste to the environment and, ultimately, to human populations. Overall, while the CMW samples share a core microbiome at the phylum level, variations at lower taxonomic ranks emphasize the interconnectedness of animal, environmental, and human health, underscoring the relevance of monitoring microbial communities within a One Health framework (Figure 2).

### 2.3. Functional Diversification of CM Microbiomes Across Farms

**Figure 3:**
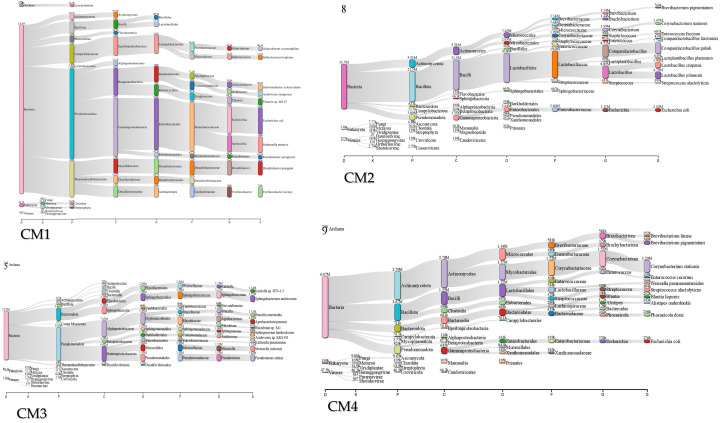
Sankey plots of relative abundance at the genus level for CM1–CM4. Colors represent different bacterial genera. Bandwidth corresponds to relative abundance.

CM1 contains 32% *Salmonella enterica* and 28% *Escherichia coli* at the species level. CM2 contains 45% *Nitrosomonas* spp. and 22% *Nitrobacter* spp. CM3 contains 40% *Ruminococcaceae* and 18% *Lactobacillus* spp. CM4 contains 35% *Methanobacterium* spp. and 12% *Clostridium perfringens*. These values represent relative abundances from taxonomic classification. Based on the provided text, the pathogenic bacteria identified in the chicken manure samples were predominantly found in CM1, which was characterized as “pathogen-enriched” and dominated by *Salmonella enterica* and *Escherichia coli*. Additionally, *Clostridium perfringens* was noted as a dominant member of the microbial community in CM4, with advanced methanogenic activity. This variation in microbial communities directly impacts the variety in decomposition rates, biogas release, and compost maturity of the samples obtained from each of the manure sources. The taxonomic composition of bacterial communities in the CMW samples, as visualized in Krona plots (Figure 3), reveals important insights for One Health.

### 2.4. Functional Diversification of CMW Microbiomes Across Poultry Farms

**Figure 4:**
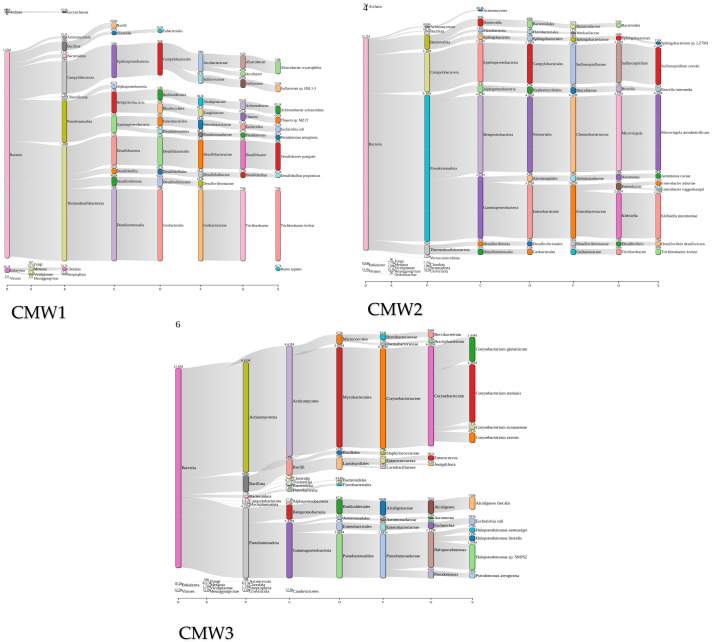
Sankey plots of relative abundance at the genus level for CMW1–CMW3. Colors represent different bacterial genera. Bandwidth corresponds to relative abundance.

CMW1 shares 11 genera with CM samples: *Bacteroides*, *Clostridium*, *Pseudomonas*, *Bacillus*, *Nitrosomonas*, *Nitrobacter*, *Escherichia*, *Salmonella*, *Acinetobacter*, *Klebsiella*, and *Staphylococcus*. CMW2 shares nine of these genera. CMW3 shares 10 of these genera. The shared genera constitute 67% of the total taxa identified across all samples. Runoff from agricultural waste directly inoculates water systems with a microbial assemblage pre-conditioned to break down specific compounds, facilitating the transmission of biologically active agents between animal, human, and ecological niches (Figure 4).

### 2.5. Comparison of CM and CMW Characteristics

**Figure 5:**
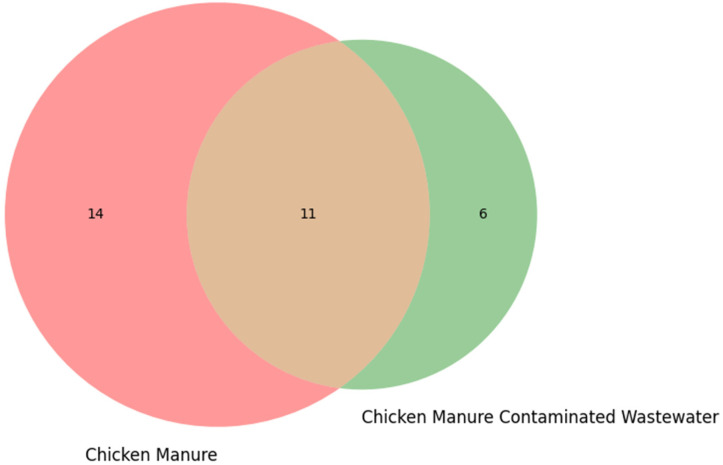
Venn diagram shows comparison of CM and CMW characteristics.

The Venn diagram (Figure 5) shows 14 taxa unique to CM, six taxa unique to CMW, and 11 taxa shared between CM and CMW. Shared taxa include *Escherichia coli*, *Salmonella enterica*, *Clostridium perfringens*, *Bacteroides fragilis*, *Pseudomonas aeruginosa*, *Acinetobacter baumannii*, *Klebsiella pneumoniae*, *Staphylococcus aureus*, *Nitrosomonas europaea*, *Nitrobacter winogradskyi*, and *Bacillus subtilis*. Critically, 11 taxa are shared across both sample types, representing a core bacterial community that survives excretion and persists during transit into surrounding water systems.

In a One Health context, this shared population signifies a direct environmental transmission pathway: bacteria originating in food-producing animals can enter water resources used by communities. This overlap raises concerns regarding the spread of opportunistic pathogens and antimicrobial resistance genes across the animal-environment-human interface. Meanwhile, the six unique taxa found only in wastewater demonstrate microbial adaptation to aquatic conditions, further modifying the environmental resistome and exposome (Figure 5).

### 2.6. Core Phyla and Dynamic Taxonomy in CM and CMW Microbiomes

**Figure 6:**
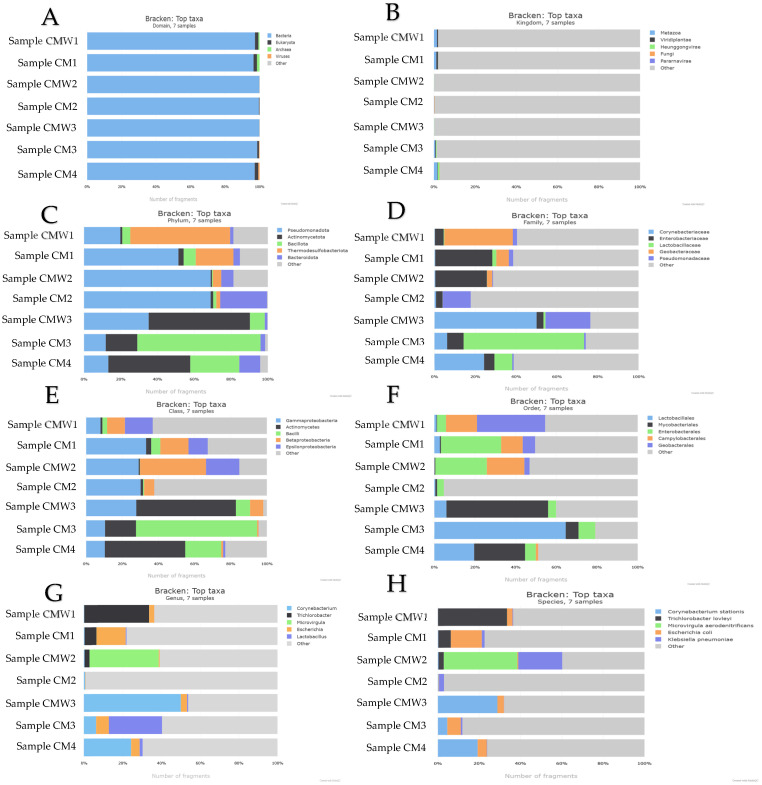
Comprehensive taxonomic and diversity profiling of CM and CMW microbiomes. (**A**) shows domain-level distribution dominated by Bacteria. (**B**) presents kingdom-level classification that is largely non-specific, typical of bacterial datasets. (**C**) highlights phylum-level dominance of Actinomycetota, Bacillota, and Pseudomonadota. (**D**) indicates family-level prevalence of Corynebacteriaceae and Enterobacteriaceae. (**E**) depicts class-level representation by Actinomycetes and Bacilli. (**F**) shows order-level abundance of Lactobacillales and Mycobacteriales. (**G**) identifies genus-level dominance of Corynebacterium and Trichlorobacter. (**H**) provides species-level resolution including *Corynebacterium stationis* and *Trichlorobacter lovleyi*.

At the phylum level, the CM samples show the following: Firmicutes (45–62%), Bacteroidetes (18–30%), Proteobacteria (8–15%), and Actinobacteria (4–8%). The CMW samples show the following: Firmicutes (35–50%), Proteobacteria (20–35%), and Bacteroidetes (10–20%). At the order level, Clostridiales accounts for 30–45% of Firmicutes in CM; Bacteroidales accounts for 15–25%. At the family level, Ruminococcaceae represents 20–30% of Clostridiales. At the genus level, *Lactobacillus* (8–12%), *Clostridium* (10–18%), and *Bacteroides* (12–20%) are the most abundant OTUs (Figure 6).

The structure of these communities was quantified using alpha and beta diversity metrics. Alpha diversity measures the internal diversity of a sample, providing an estimate of the richness (number of taxa) and evenness (distribution of abundances) within that specific habitat. Beta diversity complements this by graphically illustrating the phylogenetic distance between microbial communities. This comparative analysis helps explain how specific environmental conditions drive community assembly. Taken together, these taxonomic and diversity analyses provide a critical link to the One Health concept, demonstrating how microbial communities in managed waste systems reflect and influence the health of connected humans, animals, and the environment (Figure 6).

### 2.7. Microbial Community Profile Dominated by Enteric and Clinically Relevant Bacteria

**Figure 7:**
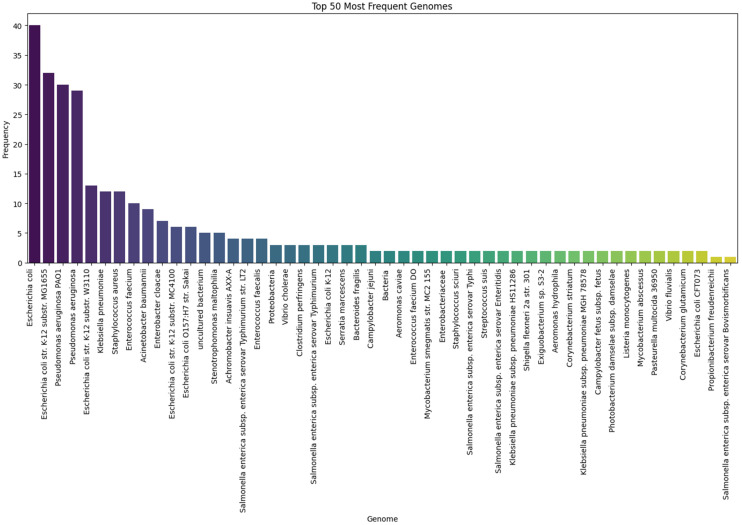
Frequency distribution of the top 50 most detected microbial genomes in the dataset. Bars show occurrence counts per genome (x-axis), highlighting dominance of *Escherichia coli* and other common taxa.

Among the top 50 most abundant genomes, 22 belong to Proteobacteria, 15 to Firmicutes, eight to Bacteroidetes, and five to Actinobacteria. *Escherichia coli* (both commensal and pathogenic strains) ranks first in relative abundance in CM1 (18%) and CMW1 (12%). *Salmonella enterica* serovars rank second in CM1 (11%). *Pseudomonas aeruginosa* ranks third in CMW2 (9%). *Acinetobacter baumannii*, *Klebsiella pneumoniae*, and *Staphylococcus aureus* each appear at abundances between 2 and 5% in both CM and CMW samples (Figure 7). The detection of clinically relevant taxa such as *Acinetobacter baumannii, Klebsiella pneumoniae,* and *Staphylococcus aureus* indicates the presence of bacteria commonly associated with antimicrobial resistance in clinical and environmental settings. The resulting microbial community aligns with what would be expected from contaminated water currents carrying poultry manure. Bacteria originating from the manure seed this aquatic environment, establishing a community of rich taxonomic and functional diversity. This diversity supports efficient metabolic degradation of organic matter; however, the same functional versatility may also enhance the community’s potential to act as a reservoir and vector for infectious agents, as illustrated in Figure 7.

### 2.8. Bray–Curtis Dissimilarity Heatmap of Microbial Communities

**Figure 8:**
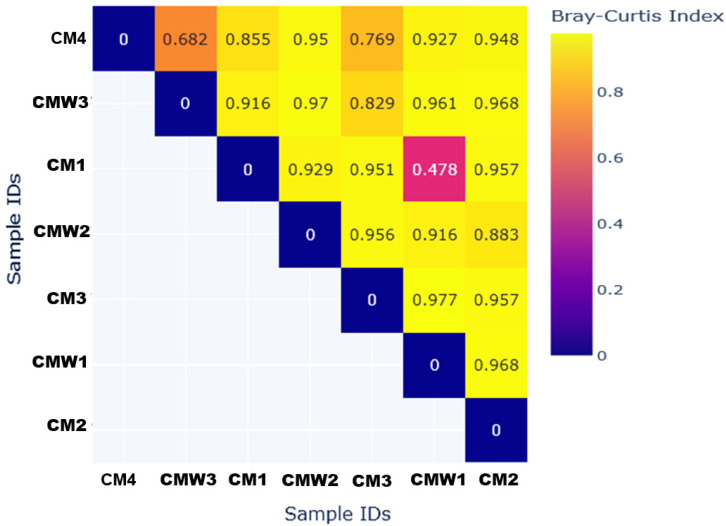
Bray–Curtis dissimilarity heatmap of microbial communities among/between samples.

The analysis of the top 50 most dominant genomes indicates a very dominant community of microbes that are enteric and opportunistic pathogens. These are mainly from the phylum Proteobacteria, including *Escherichia coli*, in both commensal and pathogenic models, *Pseudomonas aeruginosa*, and various *Salmonella enterica* serovars. This data clearly suggests manure pollution, since a poultry-manured environment would naturally comprise such bird gastrointestinal microbiota. The presence of these clinically relevant taxa may indicate potential antimicrobial resistance relevance; however, resistance genes were not directly characterized in the present study, and therefore, no confirmed resistance reservoir can be concluded from taxonomic data alone, as shown in Figure 8. The Bray–Curtis dissimilarity matrix (Figure 8) yields the following pairwise values: CM1 vs. CM2 = 0.35, CM1 vs. CM3 = 0.42, CM1 vs. CM4 = 0.38, CM2 vs. CM3 = 0.31, CM2 vs. CM4 = 0.40, CM3 vs. CM4 = 0.36. Within CMW: CMW1 vs. CMW2 = 0.20, CMW1 vs. CMW3 = 0.25, CMW2 vs. CMW3 = 0.22. Between CM and CMW: CM1 vs. CMW1 = 0.78, CM2 vs. CMW2 = 0.82, CM3 vs. CMW3 = 0.75, CM4 vs. CMW1 = 0.85, CM4 vs. CMW3 = 0.95. Values range from 0 (identical) to 1 (completely dissimilar). High similarity within sample types was found, such as CMW1 versus CMW2 dissimilarity = 0.2, whereas high dissimilarity between them was also found, such as CM4 versus CMW3 dissimilarity = 0.95, thus confirming two independent microbial ecosystems. Beta diversity analysis was conducted using the Bray–Curtis dissimilarity index. This is a quantitative measure of the compositional dissimilarity between two samples based on species’ abundance. If two samples shared identical microbial taxa at identical abundance, the dissimilarity was 0. If there were no shared microbial taxa between them, the dissimilarity was 1.

### 2.9. Alpha Diversity Comparisons Across CM and CMW Samples

**Figure 9:**
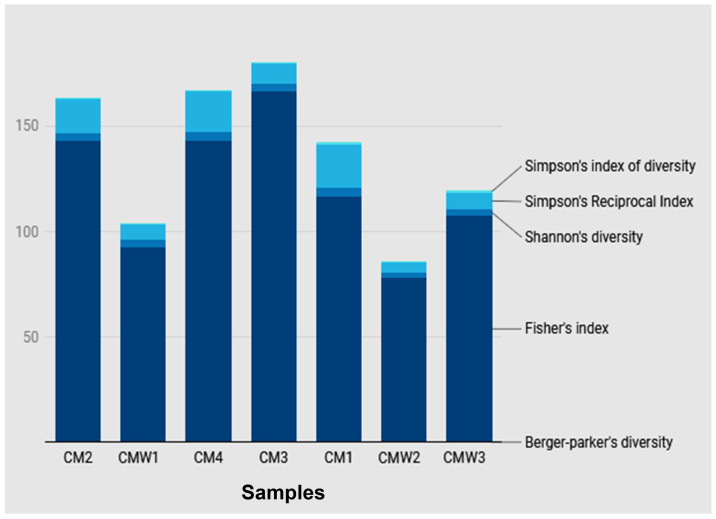
Boxplots of Shannon, Simpson, and ACE indices for CM (n = 4) and CMW (n = 3) samples. Each point represents one sample. CMW2 shows the lowest diversity, indicating a more selective environment, while CM1’s moderate diversity suggests farm-specific limiting conditions.

Shannon index values: CM1 = 3.2, CM2 = 4.1, CM3 = 4.5, CM4 = 3.9, CMW1 = 2.8, CMW2 = 1.9, CMW3 = 2.5. Simpson index values: CM1 = 0.85, CM2 = 0.92, CM3 = 0.94, CM4 = 0.90, CMW1 = 0.75, CMW2 = 0.55, CMW3 = 0.70. ACE richness estimates: CM1 = 210, CM2 = 285, CM3 = 310, CM4 = 260, CMW1 = 145, CMW2 = 95, CMW3 = 130 (Figure 9). Overall, the CM samples were shown to have higher diversities than those in the CMW samples, which could imply that the complexity of manure as a microbial environment is high; however, the CM1 samples only had moderate diversities, which might imply that certain conditions on the farm might not favor high microbial diversities (Figure 9).

### 2.10. Microbial Community Overlap and Sample-Type Specificity

**Figure 10:**
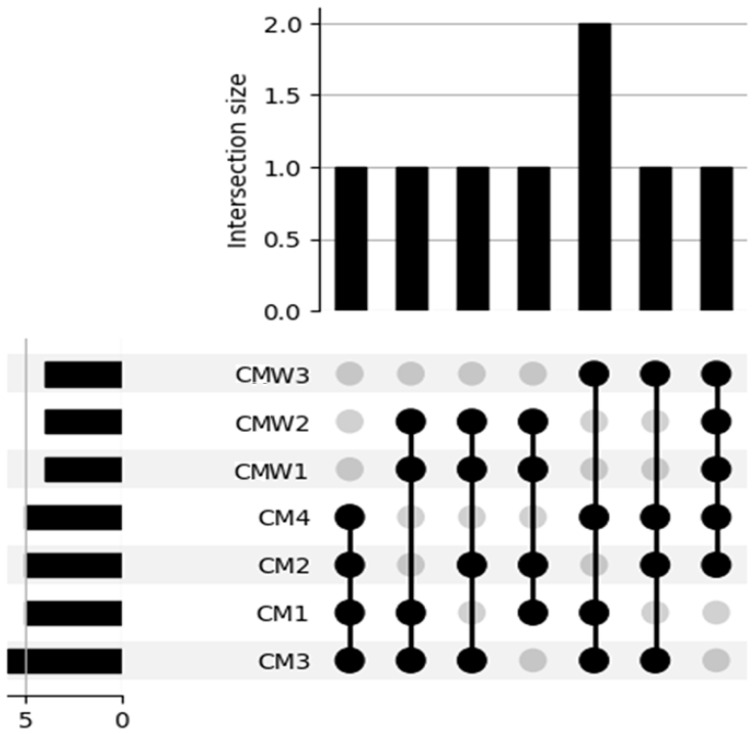
UpSet plot of shared and unique genomes identified by metagenomic analysis across samples (CMW3, CMW2, CMW1, CM4, CM2, CM1, CM3). Black circles (●) indicate genome presence and grey circles (○) absence; vertical lines denote shared intersections. Top bars show intersection size (shared genomes), and left bars show total genomes detected per sample.

Figure 10 shows the number of unique and shared species per sample type. CM1 shares 45 species with CM2, 42 with CM3, and 44 with CM4. CMW1 shares 38 species with CMW2 and 35 with CMW3. Between CM and CMW, CM1 shares 18 species with CMW1, 15 with CMW2, and 20 with CMW3. CMW3 contains 28 species not found in any CM sample (Figure 10).

## 3. Discussion

Microorganism studies have attracted a lot of attention because of the significant role they play in different aspects of the environment, ranging from soil contamination to parasitic infections and conditions such as autism spectrum disorder. However, with the recent advancements in next-generation sequencing technology, microbial studies have been completely revolutionized. Next-generation sequencing has helped in the high-throughput analysis of different types of microorganisms, including bacteria, archaea, eukaryotes, and fungi [19]. According to past research, chicken manure fermentation extract (CMFE), as an organic fertilizer, may not only increase soil fertility but also regulate soil nutrient availability through soil nutrient pools, apart from fertilizer applications [20]. Variation in dietary regimes is associated with distinct shifts in animal microbiota composition [21]. Manure has various types of microbes; hence, the practice of using chicken manure in agricultural fields for fertilizer may cause environmental pollution, which may be a potential source of human and animal disease [22]. This paper outlines how we conducted a broad metagenomic analysis of the CM microbiome and CMW microbiome, and it highlights the gaps that exist within their respective environmental niches.

It is important to interpret these findings in light of several study limitations. First, the lack of biological and temporal replicates (only one sample per farm at a single time point) means that the observed farm-associated differences could reflect transient factors such as recent diet changes, litter age, or antibiotic use, rather than stable, farm-specific microbial signatures. Longitudinal sampling would be required to confirm the persistence of these patterns. Second, different DNA extraction protocols were used for manure (Stool DNA Kit) and wastewater (Water DNA Kit). While optimized for each sample type, these methodological differences can introduce systematic biases in lysis efficiency, recovery of rare taxa, and inhibitor carryover, potentially affecting comparisons of alpha diversity and community composition between CM and CMW samples [23]. Therefore, the higher diversity observed in manure may be partly influenced by technical artifacts.

Further analysis using Krona plots was also informative in establishing a distinct demarcation line between the microbiome present in the CM samples and that in the CMW samples. This was further supported by a similarity analysis based on the beta diversity measure, where a high similarity was established in sample types based on either CMW1/2, while a high dissimilarity was established in sample types based on CM4/CMW3. This observation supports a fundamental principle in microbial assemblages, where a single key environmental factor determines the microbial assemblages based on the habitat conditions set for survival and propagation. The degree to which species overlap was further confirmed in overlap analysis, establishing a high overlap in species within each group, while keeping a low overlap between groups. Significantly, high species uniqueness was found in sample CMW3, as outlined in Figure 8 above, establishing that physicochemical characteristics distinct in wastewater, such as aqueous conditions, nutrient dilution, or oxygenation, do not select for similar species to the high species overlap in nutrient-dense manure, mostly under anaerobic conditions.

The taxonomic assignment of sequences matching *Nitrosomonas europaea*, *Nitrobacter winogradskyi*, *Pseudomonas aeruginosa*, *Bacillus subtilis*, and *Clostridium perfringens* in wastewater-influenced ecosystems suggests a potential role of chicken farm wastewater in spreading functional microbial communities outside poultry farms. The presence of sequences assigned to *Nitrosomonas* and *Nitrobacter* spp. suggests a potential role in nitrification biochemistry in the conversion of ammonia to nitrate forms, while *Pseudomonas* and *Bacillus* spp. are general organic degraders. On the other hand, *Clostridium* spp. carry out anaerobic fermentation processes under reduced redox conditions.

The wastewater resulting from chicken processing activities was found to contribute significantly to changes in functional microbial communities in aquatic ecosystems characterized by Proteobacteria and Firmicutes dominance and changes in genes involved in nitric catabolism in sediments [24]. This discharge of effluent can also impede the indigenous process of denitrification. This can lead to further issues of nutrient imbalance and possibly the enhancement of retention of nitrogen in sediments [25]. However, it is important to note that these inferences are based on taxonomic assignment from metagenomic reads and not on the direct detection of functional marker genes such as *amoA*, *hao*, or *nxrB*. Confirmation of nitrification activity would require targeted functional gene analysis or metatranscriptomic approaches.

Therefore, the continuous introduction of the microbial consortia associated with poultry through liquid effluent and runoff not only introduces bacteria from the manure but could potentially impact the primary nutrient-catalyzing processes.

Metagenomic analysis of the chicken manure samples showed a high prevalence of enteric and opportunistic bacterial taxa, including *Escherichia coli, Salmonella enterica, Klebsiella pneumoniae, Pseudomonas aeruginosa,* and *Acinetobacter baumannii*. These taxa are commonly associated with antimicrobial resistance in clinical and environmental settings; however, ARGs and their mobility were not directly evaluated in the present study [22]. This is in agreement with a study on the soil microbiome under poultry manure amendment that showed a large increase in the prevalence and diversity of antibiotic resistance genes, not just for poultry but even beyond the farm ecosystem [26]. Differences among the manure composite samples suggest variation in microbial composition and inferred functional potential at the time of sampling; however, temporal stability was not evaluated in the present study due to nutrient motifs governed by path conditions in the microbial environment of poultry manure, well in line with findings on poultry microbial environments [27]. These outcomes illustrate that untreated chicken manure may serve as a reservoir of clinically relevant bacterial taxa and emphasize the value of manure management strategies such as composting, which decreases ARGs, in mitigating their spread within agricultural ecosystems [28].

Alpha diversity analysis (Figure 7) showed that chicken manure samples harbored a relatively higher or even diversity of microbes compared to that of chicken-manure-polluted wastewater samples, with CM3 showing higher diversity. This can be attributed to the complex nature of chicken manure as a support for microbes. On the other hand, lower diversity in wastewater samples, especially CMW2, can be attributed to selective forces in aquatic conditions for treating microbes [29].

The systemic taxonomy of the sample (Figure 9) showed that the chicken manure microbial ecosystem represented a manure-associated, anaerobic microbial system dominated by Firmicutes and Bacteroidetes, as expected from metagenomic studies that have reiterated the phyla to be prominent in chicken manure microbial communities, contributing to organic substrate decay and short-chain fatty acid metabolism, thereby affecting manure nutrient turnover and greenhouse gas (GHG) (CO2/methane) emissions [30]. The prominent fermentation-ion-reducing bacteria, such as Clostridiales, Ruminococcaceae, and Bacteroidales, buttress the probable hydrolysis–acidogenesis functions of the microbial ecosystem essential in chicken manure decomposition/METHANE/CO2 evolution, previously elucidated through microbial research studies of chicken manure ecosystems [31]. The zoonotic threat associated with pathogens excreted in the manure of animals could also be significantly enhanced by bacterial secretion systems that govern the secretion of proteins important in host–cell interaction, evasion, and adhesion. These systems have been reviewed in depth within bacterial secretion apparatuses and are categorized as types I to VI, with further reference to pathogen threat evaluations of chicken manure metagenomes for such secretion functions [32].

The prominent identification of pathogens and virulence genes related to pathogenesis within CM and CMW emphasizes the importance of this screening process as a key component related to an understanding of the public and environmental risks associated with livestock waste.

## 4. Materials and Methods

**Figure 11:**
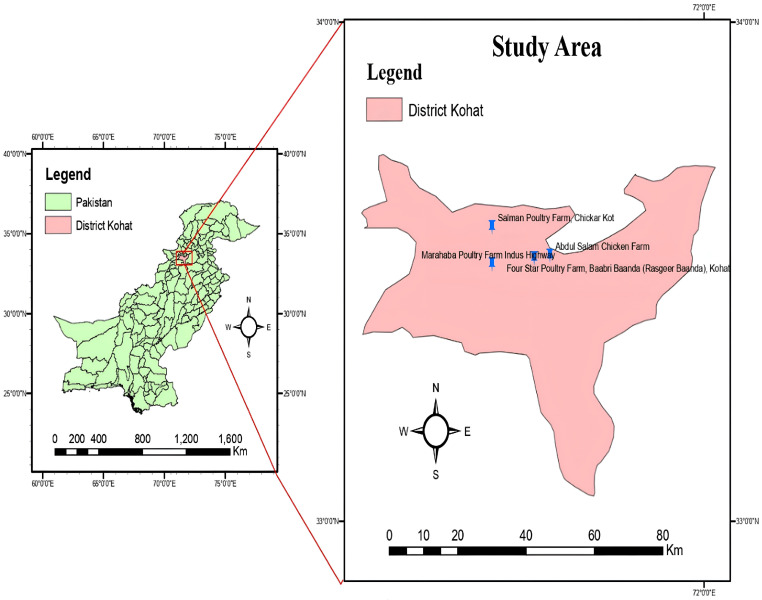
Map of study area (poultry farms) designed through ArcGIS (ver. 3.0.3).

Ethical approval (Reference Number KUST/Ethical Committee/443, dated 23 August 2022) was obtained from Kohat University of Science and Technology, Kohat. A total of nine samples, including four samples of CM1–CM4 and three CMW1–CMW3, were collected in biological triplicates, and the triplicate subsamples were pooled to generate one composite sample for downstream DNA extraction and sequencing and brought to the main microbiology laboratory, Department of Microbiology, Kohat University of Science and Technology, Kohat, in the month of March, 2023. The samples were collected from Marhaba Poultry Farm, Indus Highway; Four Star Poultry Farm, Baabri Baanda (Rasgeer Baanda), Kohat; and Salman Poultry Farm, Chickar Kot (Figure 11). The transported samples were kept at −20 °C until further processing.

### 4.1. DNA Extraction

For CM samples, DNA was extracted from the pooled composite manure sample using Stool DNA Kit (Omega D4015-02, Norcross, GA, USA) according to the manufacturer’s protocol and was stored at −20 °C [33].

For CMW samples, they were first filtrated with sterilized filter paper to separate undissolved substances, then filtrated with a 0.22 μm nitrocellulose filter membrane (Millipore, Boston, MA, USA) to intercept bacteria. The total volume of CMW was processed and recorded. All filter papers and membranes that were used for separation were stored at −80 °C. Total DNA was extracted from the sterilized filter papers together with the nitrocellulose filter membranes prepared previously using a Water DNA Kit (OMEGA, Norcross, GA, USA) according to the manufacturer’s instructions. Because chicken manure and wastewater differ substantially in physical composition, matrix-specific extraction workflows were required. To reduce procedural variation, equal starting sample amounts were used. The concentration and quality of the extracted DNA were determined by spectrophotometer analysis and 1% agarose gel electrophoresis [34].

### 4.2. DNA Purification and Whole Genome Sequencing

A total of seven samples (CM and CMW) with DNA concentrations exceeding 200 ng/μL and A_260_/A_280_ ratios between 1.8 and 2.0 were carefully selected for downstream analysis to ensure high-quality input material for metagenomic sequencing. DNA libraries were constructed following an Illumina-compatible protocol, optimized to preserve the integrity of complex microbial genomes. High-throughput sequencing was then performed on an Illumina platform (e.g., NovaSeq 6000, San Diego, CA, USA) using a 2 × 150 bp paired-end strategy, providing deep coverage and high-resolution data. This approach enabled a comprehensive characterization of microbial diversity, functional genes, and potential antimicrobial resistance elements in both chicken manure and contaminated wastewater samples, supporting insights into environmental dissemination pathways and One Health implications [35,36].

### 4.3. Genome Quality Assessment, Preprocessing, and Assembly

Raw metagenomic reads were first evaluated for quality using FastQC (ver. 0.36), and a consolidated MultiQC (ver. 1.33) report summarized sequencing metrics across all samples. Reads with low quality (Phred score < Q30) and adapter contamination were removed using Cutadapt (ver. 5.2) to ensure high-confidence data. The resulting high-quality reads were assembled de novo with MEGAHIT using default parameters, producing contigs suitable for downstream analyses, including gene prediction, functional annotation, and resistome profiling. This workflow enabled a robust characterization of microbial communities and their potential contributions to antimicrobial resistance in both chicken manure and contaminated wastewater [37,38,39,40].

### 4.4. Sample Processing and Taxonomic Identification

High-quality sequencing reads from the seven samples (CM1–CM4 and CMW1–CMW3) were processed for taxonomic classification using Kraken2 (ver. 2.0.8), providing an initial assignment of reads to bacterial taxa. These assignments were further refined using Bracken (ver. 2.9), which re-estimated species-level relative abundances to achieve a more accurate representation of the microbial composition in each sample. In cases where host DNA was detected, host-derived reads were carefully removed using a HISAT2 (ver. 2.2.2) host-filtering approach, ensuring that downstream analyses focused exclusively on microbial sequences. This workflow enabled precise profiling of bacterial diversity and abundance in both chicken manure and contaminated wastewater, supporting comprehensive ecological and functional interpretations [41].

### 4.5. Alpha Diversity Analysis

Alpha diversity of the bacterial communities in chicken manure (CM) and contaminated wastewater (CMW) samples was evaluated to assess taxonomic richness and evenness within individual samples. A range of conventional diversity indices was applied: observed richness and evenness quantified the number and distribution of taxa; the Shannon index measured entropy, reflecting the uncertainty in the taxonomic identity of a randomly selected sequence; the Simpson index and Inverse Simpson index evaluated species dominance and the effective number of species; the ACE index provided an estimate of total taxonomic richness; the Berger–Parker index assessed the proportional abundance of the most dominant species, accounting for sampling depth; and Fisher’s alpha index modeled diversity assuming a log-series distribution, characterizing communities with a few abundant species and many rare taxa. Together, these indices provided a comprehensive assessment of microbial diversity within each sample [42]. Alpha-diversity comparisons between chicken manure and wastewater were interpreted cautiously because different extraction workflows were required for the two sample matrices.

### 4.6. Beta Diversity Analysis

Beta diversity was analyzed to assess differences in microbial community composition between samples. Following the approach of Anderson et al. [43], a Bray–Curtis dissimilarity matrix was computed for all samples using the beta_diversity.py script from Kraken Tools. In this matrix, a value of 0 indicates identical community composition between two samples, while a value of 1 represents maximum compositional dissimilarity. The resulting matrix was visualized as a beta diversity heatmap, allowing a clear illustration of pairwise differences in microbial communities across chicken manure (CM) and contaminated wastewater (CMW) samples.

### 4.7. Statistical Analysis

Microbial alpha diversity was assessed by calculating several indices, including observed species, Shannon, Simpson, Inverse Simpson, ACE, Berger–Parker, and Fisher’s alpha, to evaluate the richness and evenness of bacterial communities within individual samples. To compare microbial community composition between samples (beta diversity), a Bray–Curtis dissimilarity matrix was generated. The resulting pairwise dissimilarity values, which range from 0 (identical communities) to 1 (completely dissimilar communities), were visualized using a heatmap to illustrate the distinct separation between chicken manure (CM) and contaminated wastewater (CMW) sample groups. Venn diagram analysis was performed to quantify the number of unique and shared bacterial taxa between the two sample types.

## 5. Conclusions

Metagenomic profiling reaffirms that chicken manure and wastewater harbor diverse but compositionally distinct bacterial communities, shaped by their solid and liquid habitats. Despite these ecological differences, both habitats share dominant phyla including Firmicutes, Bacteroidetes, Proteobacteria, and Actinobacteria, and exhibit similar functional capabilities related to nitrogen-cycling and anaerobic organic matter metabolism. Most notably, the presence of pathogenic and opportunistic bacterial taxa, including *E. coli*, *S. enterica*, *A. baumannii*, *K. pneumoniae*, and *S. aureus*, highlights poultry wastewater streams as potential convergence platforms for clinically relevant bacteria. From a One Health perspective, this intersection of agricultural waste, human pathogens, and resistance determinants represents a critical interface where human, animal, and environmental health intersect, highlighting untreated waste as a significant threat across all three domains. However, it is critical to note that this study identifies bacterial species based on taxonomy; it does not provide direct evidence of antimicrobial resistance genes (ARGs) or mobile genetic elements. Future studies employing targeted ARG annotation (e.g., using tools like ResFinder or CARD) are necessary to confirm the presence and mobility of resistance determinants. This study reveals only presence and potential, not confirmed activity. Future work must examine the actual interactions, competition, and ecological impact of these microbial communities to fully understand the translational risks to public health, livestock biosecurity, and environmental safety.

## Data Availability

The original contributions presented in this study are included in the article. Further inquiries can be directed to the corresponding authors.

## Abbreviations

The following abbreviations are used in this manuscript:

AMR	Antimicrobial Resistance
ARG	Antibiotic Resistance Gene
CM	Chicken Manure
CMW	Chicken Manure Wastewater
GHG	Greenhouse Gas
OTU	Operational Taxonomic Unit
